# Viroporins, Examples of the Two-Stage Membrane Protein Folding Model

**DOI:** 10.3390/v7072781

**Published:** 2015-06-26

**Authors:** Luis Martinez-Gil, Ismael Mingarro

**Affiliations:** Department of Biochemistry and Molecular Biology, ERI BioTecMed, University of Valencia, Dr. Moliner 50, 46100 Burjassot, Spain; E-Mail: ismael.mingarro@uv.es

**Keywords:** viroporins, membrane insertion, helix-helix packing, transmembrane protein folding, influenza A virus M2

## Abstract

Viroporins are small, α-helical, hydrophobic virus encoded proteins, engineered to form homo-oligomeric hydrophilic pores in the host membrane. Viroporins participate in multiple steps of the viral life cycle, from entry to budding. As any other membrane protein, viroporins have to find the way to bury their hydrophobic regions into the lipid bilayer. Once within the membrane, the hydrophobic helices of viroporins interact with each other to form higher ordered structures required to correctly perform their porating activities. This two-step process resembles the two-stage model proposed for membrane protein folding by Engelman and Poppot. In this review we use the membrane protein folding model as a leading thread to analyze the mechanism and forces behind the membrane insertion and folding of viroporins. We start by describing the transmembrane segment architecture of viroporins, including the number and sequence characteristics of their membrane-spanning domains. Next, we connect the differences found among viroporin families to their viral genome organization, and finalize focusing on the pathways used by viroporins in their way to the membrane and on the transmembrane helix-helix interactions required to achieve proper folding and assembly.

## 1. Introduction

Viroporins were first identified in 1978, after the observation that a viral infection could induce changes in the permeability of the host cell plasma membrane [1]. This seminal work, and many others that followed, established the idea of viroporins as a protein family [2]. Viroporins play a key role in the virus life cycle; deletion of a viroporin-encoding gene from a viral genome drastically diminishes viral fitness. In fact, viroporins have been successfully exploited as therapeutical targets [3,4], with the influenza matrix 2 (M2) protein being the most prosperous example. The M2 channel inhibitors amantadine and rimantadine have been extensively used clinically [5], and nowadays, regardless of their current restricted use due to the emergence of resistant viruses, M2 inhibitors are still considered a promising target for anti-influenza drug discovery [6]. Furthermore, influenza A viruses lacking the M2 protein are being investigated as live attenuated influenza vaccines [7]. Besides their ability to permeabilize membranes, viroporins share other structural and functional features. They are small proteins, ranging approximately from 6 to 12 kDa, with an elevated α-helical content. Another common feature among viroporins is their elevated hydrophobicity; in fact, all viroporins described to date are considered integral membrane proteins.

Viroporin-induced membrane premeabilization depends on homo-oligomerization [8]. Therefore, viroporins must, first, reach and insert into the membrane and, second, interact with themselves to form higher ordered structures that will eventually be the functional units. This two-stage process resembles the original two-stage model proposed in 1990 by Engelman and Poppot for α-helical membrane protein folding [9]. In their original model the authors propose that folding of many integral α-helical membrane proteins can be envisioned in two subsequent stages. In the first stage, hydrophobic alpha-helices would be established across the lipid bilayer; and in a second stage, the already inserted helices would interact with each other to form higher order functional transmembrane (TM) structures.

In the present review we use the two-stage model as a leading thread to analyze not only the mechanism and forces required for the membrane insertion and folding of viroporins from RNA viruses but also the cellular machinery involved in the process *in vivo*.

## 2. Membrane Architecture

Nieva *et al.* [10] proposed a classification of viroporins (from RNA viruses) based on the number and orientation of their TM segments. Two major groups have been described: Class I and Class II, depending on whether the protein contains one or two membrane-spanning domains. Each of these two classes can be divided into two minor groups according to their membrane topology. Class IA viroporins locate the amino-terminus (Nt) in the endoplasmatic reticulum (ER) lumen and the carboxyl-terminus (Ct) in the cytoplasm. Influenza A virus (IAV) Matrix 2 protein (M2) and Human immunodeficiency virus (HIV-1) Viral Protein Unique (Vpu) are the best known examples of Class IA viroporins. Class IB viroporins have a reversed orientation, with the Nt oriented towards the cytosol and the Ct facing the ER lumen, with Human respiratory syncytial virus (HRSV) short hydrophobic protein (SH) falling into this category. In Class IIA, both Nt and Ct are located within the ER lumen (the Hepatitis C virus (HCV) protein 7 (p7) and Sindbis virus (SINV) 6 kDa protein (6K) are examples of this class). Poliovirus 1 (PV1) 2B, one of the most studied viroporins and the only described member of Class IIB, adopts the opposite orientation with both ends facing the cytosol. Interestingly, Class II viroporins are generally produced from viral polyproteins through proteolytic processing, whilst Class I do not require cleavage to reach their mature form [10,11]. The differences between Class I and II viroporins, as we will show next, go beyond the number and orientation of the TM segments, and they might reflect a discrepancy in the mechanism of action and/or the target membrane. There are few exceptions to this classification (e.g., 3a protein of human severe acute respiratory syndrome corona virus (SARS-CoV)) has three TM segments [12]). However, in this review we focus on the already describe Class I and Class II viroporins exclusively. Nonetheless, SARS-CoV 3a requires (like any Class I or Class II viroporin) insertion into the membrane followed by homo-oligomerization to perform its pore forming activity; and the principles governing both steps (stage I and stage II) apply to all membrane proteins.

**Figure 1:**
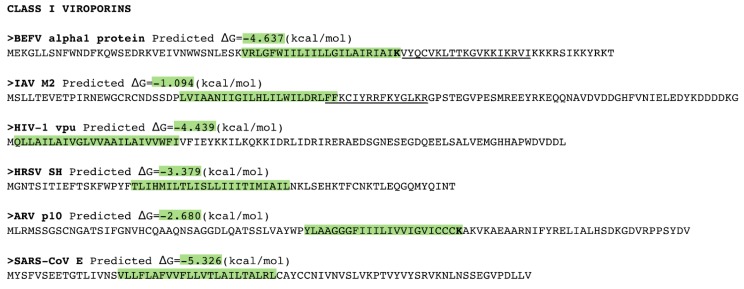
Sequence analysis of Class I and Class II viroporins. Sequence, in FASTA format, of the Class I viroporins included in this review. The putative transmembrane (TM) segments found by the ΔG Prediction Server v1.0 [13] and their predicted ΔG values (kcal/mol) are highlighted in green (indicating a negative ΔG value). The Lys residues within the TM segments are displayed in bold. Amphipathic TM flanking regions are underlined. BEFV Bovine ephemeral fever virus, IAV Influenza A virus, HIV Human immunodeficiency virus, HRSV Human respiratory syncytial virus, ARV Avian reovirus, SARS-CoV Severe acute respiratory syndrome Coronavirus.

**Figure 2:**
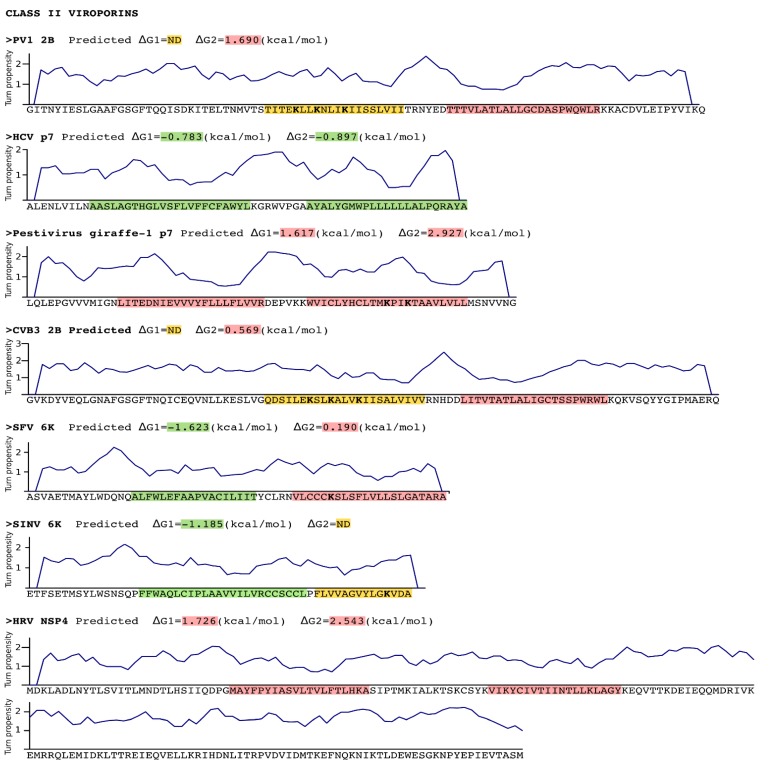
Sequence analysis of Class II viroporins. Sequence, in FASTA format, of the Class II viroporins included in this review. The putative TM segments found by the ΔG Prediction Server [13] and their predicted ΔG values (kcal/mol) are highlighted in green (indicating a negative ΔG value) or in red (positive ΔG value). Those TM segments for which existence there are evidences (experimental or computational) but were not detected (ND) by the algorithm are highlighted in yellow. The Lys residues within the TM segments are displayed in bold. A turn propensity plot is depicted above each protein sequence. For this analysis the turn propensity at each position was calculated using the data on Monné *et al.* and a slide window of 5 amino acids. PV1 Poliovirus 1, HCV Hepatitis C virus, CVB3 Coxsackievirus B3, SFV Semliki forest virus, SINV Sindbis virus, HRV Human rotavirus A.

**Figure 3:**
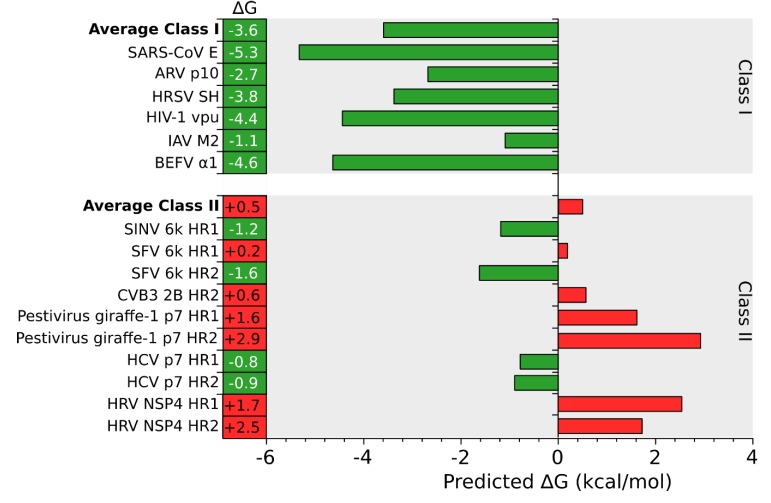
*In silico* prediction of putative TM segments. An analysis of the putative TM segments of Class I (top) and II (bottom) viroporins was done using the ΔG Prediction Server [13]. The figure shows the predicted ΔG (kcal/mol) for each putative TM segment and the average ΔG for Class I and II viroporins. Green and red bars indicate negative and positive ΔG values respectively. BEFV Bovine ephemeral fever virus, IAV Influenza A virus, HIV Human immunodeficiency virus, HRSV Human respiratory syncytial virus, ARV Avian reovirus, SARS-CoV Severe acute respiratory syndrome Corona virus, PV1 Poliovirus 1, HCV Hepatitis C virus, CVB3 Coxsackievirus B3, SFV Semliki forest virus, SINV Sindbis virus, HRV Human rotavirus A.

An *in silico* analysis of the hydrophobic profile of viroporins using the ΔG Prediction Server [13,14,15], an algorithm that predicts the presence of TM segments based on the apparent free energy difference (ΔGapp) for the insertion of a putative TM helix into the ER membrane by means of the Sec61 translocon, identifies for class I viroporins a single TM segment with highly favorable ΔG values (−3.59 kcal/mol in average ranging from -1.09 to -5.33 kcal/mol for the analyzed sequences) (Figure 1, Figure 2 and Figure 3). On the other hand, for class II viroporins the algorithm detects, not one but two TM segments with a surprisingly positive ΔGapp value (+0.5 kcal/mol in average). Furthermore, in some cases (eg. 2B from PV1) only one TM helix is predicted; the other hydrophobic region is not recognize as a TM by the algorithm, probably due to its low hydrophobicity, even though its presence has been experimentally confirmed [16].

In class II viroporins the TM segments are connected by a short stretch of amino acids (1–8 residues) forming what is known as an α-helical hairpin. This motif is thought to occur relatively frequently in integral membrane proteins and may serve as an important structural and/or functional element. The close proximity between both TM segments in an α-helical hairpin facilitates interactions between them. These interactions are, precisely, what may allow the insertion in the membrane of marginal hydrophobic TM segments (see below). In fact, it has been demonstrated that insertion of the PV1 2B viroporin α-helical hairpin requires an electrostatic interaction between amino acid residues located in both TM helices [17]. This might not be a unique feature of the viroporins α-helical hairpins; naturally occurring helical hairpins are in many cases not highly hydrophobic [18]. This type of interactions would require: 1. A concerted mechanism of insertion (see *Insertion into the ER membrane* section) since interactions must form prior to partition of the α-helical hairpin into the membrane. 2. The region connecting the TM segments must grant the formation of the hairpin by promoting a turn. In the case of the PV1 2B it was shown that the α-helical hairpin is stabilized by a short loop heavily populated by turn-promoting residues [17]. Again, this is not a unique feature of the 2B viroporin. A turn propensity analysis (using the scale described by Monné et al. [19] and a slide window of 5 amino acids) reveals an area with an elevated turn propensity between the putative TM segments of most class II viroporins (Figure 2).

## 3. Stage I: Protein Insertion into the Membrane

### 3.1. Targeting the ER Membrane

Viroporins are located, mainly in the internal membrane systems (ER and Golgi) or at the plasma membrane [10]. However, HCV p7 and PV1 2B have recently been found, to a minor extent, associated with the mitochondrial membrane [20,21]. In this review we will focus, exclusively, on the pathways that leads to the insertion of viroporins into the mammalian ER membranes and those ER-derived membranous organelles. For complete reviews on mitochondrial protein biogenesis see: [22,23,24]. However, the principles and forces that govern TM helix-helix packing are expected to be the same in all membranous compartments including the mitochondria.

Correct protein localization is critical for any living organism. The pathway that a protein uses to reach its final destination depends, primaryly, on the interactions of the polypeptide nascent chain once it emerges from the ribosome exit tunnel. Most cellular membrane proteins encode in their Nt a short sequence known as signal sequence (SS). In the case of ER targeting the SS consists of a continuous stretch of hydrophobic residues (6 to 20) with one or more basic residues to one side of the hydrophobic core [25]. Once the SS is outside of the ribosome exit tunnel it is recognized by the Signal Recognition Particle (SRP). The SRP will, through interactions with the ribosome, transiently stop translation [26] and subsequently dock the ribosome–nascent chain–SRP complex to the ER membrane via the SRP receptor (SR) [27]. The ribosome with the nascent chain is then transferred to the translocon, a multiprotein complex that facilitates polypeptide translocation across and insertion into the ER membrane. Upon SRP disengage the translation re-starts and the insertion into the membrane proceeds through the translocon. In this case, the translocation/insertion of the nascent chains occurs while the protein is being translated, that is to say, co-translationally. Alternatively, in the absence of a cleavable SS, the SRP can recognize a TM segment (if its hydrophobicity, length and location within the protein sequence are compatible with SRP requirements). In this case, the TM domain performs a dual function as a signal sequence and as membrane anchor domain. The absence of a SS is relatively common among viral membrane proteins, specially in an RNA virus where genome space is exceptionally restricted.

If there is no SS or TM segment at the Nt of the polypeptide or when the first hydrophobic domain is located far enough from the starting codon [26,28], the SRP will not be able to direct the emerging nascent chain to the SR. When this happens the protein will insert into the membrane after translation is completed (i.e., post-translationally). Post-translational targeting into the ER membrane uses cytosolic molecular chaperones [29,30] that prevent protein misfolding and keeps the newly synthesized in a translocation/insertion competent state until they reach the ER membrane. Surprisingly, the mammalian cytochrome b5 can enter the ER membrane unassisted [31], demonstrating the possibility of an alternative machinery-free insertion mechanism.

Already in the eighties it was shown that the M2 channel requires the SRP for its insertion into the membrane [32]. Since then, M2 has served as a model protein not only for class I viroporins but also for small single-spanning membrane proteins in general. M2 lacks a cleavable SS, its TM domain acts as a signal-anchor domain recruiting the SRP and subsequently directing the protein to the translocon where insertion occurs co-translationally. Due to the similar size and membrane architecture between class I viroporins (see above) it is generally assumed that all of them use the co-translational pathway in their way to the ER membrane. The different topologies found between subclasses IA and IB would arise from rearrangements of the nascent chain while locating within the translocon (see [33] for a recent review).

Unlike class I viroporins, where membrane insertion depends exclusively on their primary structure, class II viroporins are inserted in the context of the poly-protein in which they are encoded. This can lead to different situations. On one side of the spectrum we have HCV p7 viroporin. The core protein of this flavivirus, located in the 5' end of the viral genome, directs the entire polypeptide via SRP [34] into the ER membrane where insertion occurs co-translationally [35,36]. Consequently, p7 is already inserted into the lipid bilayer when it adopts its mature form after proteolytic cleavage [37]. On the other hand, PV1 polyprotein contains its first hydrophobic segment with the potential to deliver the viral polyprotein to the translocon, at position 451 (based on [14,15]). However, current data suggest that nascent chains with the hydrophobic segment in such downstream position are not directed to the membrane through the SRP and should be inserted post-translationally. In fact, no SS is predicted in PV1 polyprotein sequence (according to the Signal Sequence prediction algorithm [38,39]). However, precursor cleavage between P1 and P2 occurs during protein synthesis [40]. This auto-proteolytic activity liberates the P1 precursor and leaves a “new” protein starting residue 183 amino acids upstream the first hydrophobic domain (2B first TM segment). In this situation the SRP could recognize and bind to this hydrophobic sequence, stop translation and deliver the nascent chain into the ER membrane for a co-translational insertion. In fact, isolated PV1 2B has the potential to be inserted co-translationally into microsomal membranes when expressed *in vitro* [16]. Nevertheless, peptides derived from PV1 2B hydrophobic regions can recapitulated the membrane porating activity, even in an artificial membrane system [41], suggesting a potential post-translational insertion mechanism for these sequences. Clearly, more work needs to be done to elucidate the mechanism of insertion of PV1 2B, specially in the context of the full length genome.

### 3.2. Insertion into the ER Membrane

Most integral membrane proteins, regardless of their route to the membrane (SRP dependent or independent) or their mechanism of insertion (co-translational *vs* post-translational) use the translocon to bury their hydrophobic segments into the lipid bilayer. The role of the translocon goes far beyond the partition process itself; it discriminates between what will or will not be a TM segment, sets the topology of the protein (based on the topology determinants present in the polypeptide sequence) and facilitates coordinated insertion of multiple TM segments.

The translocon constitutes a channel between the cytosol and the ER lumen. It adopts in its closed configuration an hourglass shape with a “plug” in the narrowest point to maintain the permeability barrier of the membrane. The translocation/insertion process starts with the interaction between the translocon and its cytosolic counterpart (the ribosome in the case of the co-translational insertion pathway). This interaction triggers conformational changes in the translocon to accommodate the incoming polypeptide; a lateral ‘crack’ appears in the cytosolic side while the hourglass middle constriction increases its diameter [42,43,44]. As the polypeptide enters the channel additional structural modifications occur. First, the plug is displaced. Next, the lateral crack expands across the entire channel creating a lateral gate facing the core of the membrane [45]. At this stage the protein sequence within the translocon will, depending on its characteristics, laterally partition into the hydrophobic core of the membrane (in the case of TM segments) [33] or, if the polypeptide region is not hydrophobic enough, continue through the channel to be translocated into the ER lumen.

Membrane protein insertion is, essentially, a thermodynamic partitioning process between the aqueous environment of the cytosol and the hydrophobic core of the lipid bilayer, in which the hydrophobic profile of the polypeptide is the primary force behind its insertion. *In vivo*, the process is facilitated by the translocon which exposes the nascent chain, whose fate has not been decided yet, to the hydrophobic membrane core. The correlation between the hydrophobicity scales obtained *in vivo* [14] and *in vitro* [46] reinforces the idea that recognition of TM segments by the translocon involves direct interaction between the segment to be inserted and the surrounding lipids. Partition from the translocon channel into the membrane will depend not only on the amino acid composition but also on the precise position of the residues within the hydrophobic segment, the TM segment orientation, and the length of the helix [14,15,47,48]. Even amino acid residues flanking a putative TM domain could affect the insertion process due to their preference for the cytosolic or luminal side of the membrane [49].

The favorable free energy of partitioning a hydrophobic amino acid side chain into the membrane is opposed by the unfavorable cost of partitioning the peptide bond. However, if the peptide backbone adopts a secondary structure maximazing the formation of intra-protein-backbone hydrogen bonds, the energy penalty for the insertion will be greatly reduced [50,51,52]. Therefore, the formation of secondary structure (α-helix in the case of viroporins) is critical for membrane insertion of a TM segment. The favorable effect of hydrogen bonding is not restricted to intra-helical bonds. In multi-spanning membrane proteins the presence of inter-helical hydrogen bonds can also facilitate the insertion of an otherwise unfavorable segment [51,52,53,54]. Nevertheless, intra/inter-molecular hydrogen bonds do not eliminate completely the energy penalty associated with the insertion of a peptide bond. The cost of dehydrating the peptide bond is still elevated [55] and this must be compensated by the favorable energy of inserting a significant amount of hydrophobic side chains. This phenomenon explains the commonly elevated hydrophobicity profile of TM segments. 

As mentioned before, inter-helical hydrogen bonds and electrostatic interactions can facilitate the insertion of polar residues present in adjacent TM regions, like the ones found in PV1 2B viroporin hairpin [17]. The formation of inter-helical electrostatic interactions requires that the implicated helices move from the translocon into the membrane after the interaction takes place and at the same time, in what is known as concerted insertion. During the biogenesis of multispanning membrane proteins once the first hydrophobic segment reaches the translocon it must be relocated to accommodate the following TM domains. There are at least two options: 1. Each hydrophobic segments leaves the translocon and partitions into the membrane individually [56]. 2. Two or more TM segments accumulate in the translocon or in its proximity and partition into the lipid core in block [57,58]. It is thought that highly hydrophobic TM segments would leave the translocon individually, while less hydrophobic segments would need to interact with other helices in the translocon or its associated proteins before partitioning into the membrane [59]. In fact, interactions between TM segments with low hydrophobicity at early stages of the insertion process have been identified [60,61]. Furthermore, marginally hydrophobic TM segments are retained in the translocon longer than highly hydrophobic TM domains [62].

**Figure 4:**
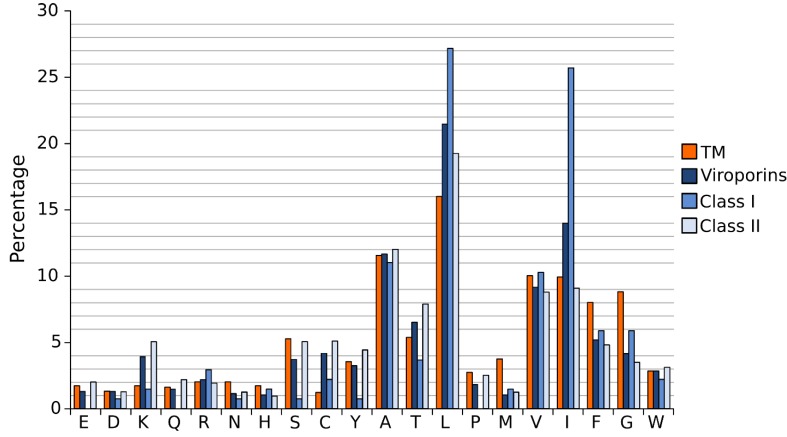
Amino acid distribution in viroporin TM segments. Amino acid distribution from 792 TM helices from a set of non-redundant proteins of known structure (orange) and viroporin putative TM regions (blue) (data from viroporins shown in Figure 1 and Figure 2). Viroporin hydrophobic regions were analyzed jointly (darkest blue) and separately by class (lighter blues).

A close analysis of the hydrophobic regions of class I viroporin reveals an over-represented number of the highly hydrophobic Leu and Ile residues when compared to other TM segments (Figure 4). Figure 4 shows the amino acid composition of a small group of class I and II viroporins TM segments (data obtained from sequences presented in Figure 1 and Figure 2) compared to TM helices from membrane protein structures (data from [18]). This unusual number of Leu and Ile residues could be a consequence of their membrane architecture. In class I viroporins the single TM domain must be able to direct and anchor the protein to the membrane by itself. No interactions with other TM segments that could facilitate the insertion are possible at this point. Therefore, class I TM domains must be hydrophobic enough (higher percentage of highly hydrophobic residues) to fulfill the process successfully. Furthermore, regardless of the protein sequence the formation of a functional water-filled channel from monomeric helical subunits requires some amphipatic distribution. Consequently, in order to achieve quaternary structures an energy penalty step should be overcomed, either at stage I or at stage II of the insertion/folding process. Either polar residues that will end up facing the aqueous environment once the channel is formed must be inserted in the non polar membrane (stage I), or a hydrophobic surface is exposed to the water rich channel interior once the monomers create the water filled channel (stage II). In any case, this energy penalty must be compensated by other forces. Probably, by the favorable insertion of an elevated number of highly hydrophobic side chains (Leu and Ile) facing the lipids [63].

Interestingly, most class II viroporins contain an elevated number of Lys residues, an amino acid residue highly penalized in TM segments (Figure 1, Figure 2 and Figure 4) [14]. From the partition point of view, this abundance is difficult to explain. However, the presence of Lys in pore forming peptides has been extensively documented [64,65,66] and, most likely, it is key for class II viroporins function. This discrepancy in Lys residue content between class II and I viroporins may, once more, highlight differences in the mechanism of action and/or the type of pore that is formed by each viroporin class. While most class I viroporins form highly regulated ion channels (see the influenza M2 example below), class II are thought to disrupt membrane permeability by creating proteolipidic pores similar to those formed by melittin or pro-apoptotic proteins such as Bax [67]. Notably, HCV p7, the only class II viroporin with a demonstrated co-translational insertion does not contain any Lys residue in its hydrophobic regions; furthermore, it is thought to form an ion channel like class I IAV M2 [8,68,69]. HCV p7 might constitute an exception within class II due to its mechanism of action/insertion or, alternatively, within class I due to the number, length and hydrophobicity profile of its TM regions. Consequently, the presence/absence of Lys residues may indicate not only a difference in the type of pore constructed but also distinct route of insertion into the membrane. In fact, these two features seem to be related.

## 4. Stage II: Transmembrane Helix-Helix Interactions

Viroporins do not work individually but coordinately. Due to their membrane embedded nature and architecture, protein-protein interactions among viroporins are thought to occur mostly through their TM segments. In order to fully understand the role of viroporins within the cell we must first identify the forces behind TM helix-helix packing. These forces are essentially the same as those driving interactions between soluble proteins. However, their contribution to the folding/packing of the protein is significantly different due to the modified environment (cytosolic *vs* lipidic). As mentioned before, in soluble proteins the tertiary and quaternary folding is mainly driven by the hydrophobic effect and electrostatic interactions. In contrast, in TM proteins van der Waals interactions have been identified as the primary force behind the helix-helix packing. One could argue that the strength of these forces/interactions is fundamentally different depending on the environment. However, that is not the case. The strength of side chain hydrogen bonds in soluble and membrane proteins is in average ~ 0.5kcal/mol and ~0.6kcal/mol respectively [70]. Similarly, the contribution of van der Waals forces to the folding of a soluble protein was estimated as 20-24 cal/mol/Å, and 18-36 cal/mol/Å for membrane proteins [71].

The formation of salt bridges and inter-side chain hydrogen bonds requires two polar amino acids with opposite polarity. These residues are extremely unfavorable within the lipid bilayer. Their presence in TM segments is significantly abated (Figure 4) [14]. As a consequence, their contribution to the folding/packing of TM segments will be limited. However, salt bridges and hydrogen bonds are not completely avoided in TM helix-helix interactions (see M2 below) [72,73]. The energy penalty of inserting a polar residue could be compensated by the presence of highly hydrophobic residues. Furthermore, if the polar interaction is formed before partition into the lipid core, which would require a concerted mechanism of insertion (see above) and a close proximity between the implicated residues (see below), the energy penalty could be greatly eliminated. Notably, when salt bridges or hydrogen bonds occur, their contribution to the folding/packing of TM segments is substantial [74].

In the non-polar membrane environment the hydrophobic effect is responsible primarily for the insertion into the membrane (stage I). All in all, once salt bridges, hydrogen bonds and the hydrophobic effect are removed from the equation the tertiary and quaternary structure of TM segments are dictated by a delicate balance of the remaining low energy forces, where van der Waals interactions play a crucial role. For a full review on the forces driving membrane protein folding see [75]. The nature of van der Waals forces requires a large contact area between the associating protein segments. Interestingly, in TM helical segments amino acids with small side chains (Gly and Ala) favor the helix-helix contact interfaces, while bulky non-polar side chains locate mostly on lipid expose surfaces [76], suggesting that intimate packing is fundamental for their association. Certainly, the role of Gly in helix-helix association has been extremely well documented in the context of Glycophorin A [77]. One could assume that viroporins would have a large number of Gly and Ala to facilitate helix packing, but this is not the case (Figure 1, Figure 2 and Figure 4). In fact, it is just the opposite. The Gly content in viroporin TM segments is lower than in other TM domains, specially for class II viroporins. There are several possible explanations for this phenomenon: 1. Viroporins, or at least some of them, do not interact directly with each other but simply work coordinately. Probably, this is the case for class II viroporins. On the other hand, the data that we have for some class I viroporins indicates a physical interaction between monomers. 2. Interactions occur through other domains (extramembranous regions) of the proteins. This second option does not seem feasible for some viroporins. Short peptides derived from PV1 2B and Classical swine fever virus (CSFV) p7 (both class II viroporins) TM segments can permeabilize membranes (no complementation with the soluble domains is required either in cis or trans) [41,78]. Then, if the interaction is required for the activity, the TM segment is responsible for it. Furthermore, in the case of IAV M2 (see below) and HCV p7 [79] structural studies show that the isolated TM segment is sufficient for tetramer or hexamer formation respectively. On the same lines, computational work on HIV Vpu and IAV M2 suggests that the TM segment (in both cases) is sufficient for helix assembly [80,81]. 3. There is a third possibility in which the TM segment is responsible for the interaction among proteins but where van der Waals forces are not the main contributor (see M2 below). It is worth mentioning that in the case of viroporins (at least in class I) the packing must be loose enough to allow the formation of an aqueous channel that facilitates ion passage through the pore. In this scenario small residues (like Gly) that promote a close packing might not be favored.

The membrane architecture (which in part depends on the route of insertion co- *vs* post-translational) will, undoubtedly, determine the interaction possibilities of the TM segment. Therefore, differences between class I and class II TM domains will probably drive these two porating families into a different mechanism of interaction and subsequently into a different mechanism of action.

## 5. Influenza Matrix Protein 2: Example of Quaternary Structure 

The M2 structure has been extensively investigated, and it constitutes an excellent example to explore TM segment interactions of viroporins. However, the conclusions obtained from M2 might not be representative of all viroporins. As we have argued previously, Class I and Class II might act in completely different manners. Furthermore, differences in the sequence within Class I viroporins could reflect variations in the mechanism of action.

The influenza A virus is a negative sense RNA virus form the Orthomyxoviridae family. Its genome comprises eight RNA segments. Segment seven codes for the matrix 1 protein (which forms the viral protein coat) and by RNA splicing the M2 protein [82]. Following attachment (via the hemmagglutinin protein) the virus is endocytosed. The acidity of the endosomal compartment triggers two crucial processes. First, the low pH promotes a conformational change in HA, exposing the fusion peptide and subsequently the merging of the viral envelope with the endosomal membrane. Second, protons from the endosome are transfered into the virus via the M2 channel. Internal acidification of the influenza A virion is responsible for disruption of the viral coat protein-protein interactions, allowing the viral genome to be released into the host cytoplasm. Later in the life cycle, the M2 channels counteracts the acidity of vesicular compartments of the exocytic pathway in infected cells protecting the structural integrity of the acid-sensitive glycoprotein [83]. M2 viroporin also promotes budding of the mature virus from the cell surface by the active generation of negative Gaussian membrane curvature [84]. In all these processes the correct formation of M2 quaternary structure is essential.

The protein can be divided in four functional regions (Nt to Ct): 1. An extracellular unstructured short domain (residues 1-24) involved (in the case of influenza A) in the incorporation into the virion [85]. 2. A TM domain, residues 25-46. This region is responsible for both tetramerization and proton conductance [86]. 3. A cytoplasmic membrane proximal amphiphilic helix involved in budding, scission and membrane localization [84,87]. 4. A Ct disordered tail necessary for M1-M2 interaction [88].

The M2 protein exist in a monomer-tetramer equilibrium (a perfect example of the two-stage model). Before the resolution of the protein structure, the TM domain was already identified as responsible for the channel tetramerization [89] and proton transport activity [90]. There has been some controversy on the role of the cytoplasmic amphiphilic helix in both processes. However, several studies showed that mutations/deletions of the membrane proximal helix had no effect on surface expression levels or proton transport; the only change being its inability to promote membrane scission and virion release [87,91]. All in all, these results show the M2 TM domain as necessary and sufficient for tetramer formation and proton conductance.

**Figure 5:**
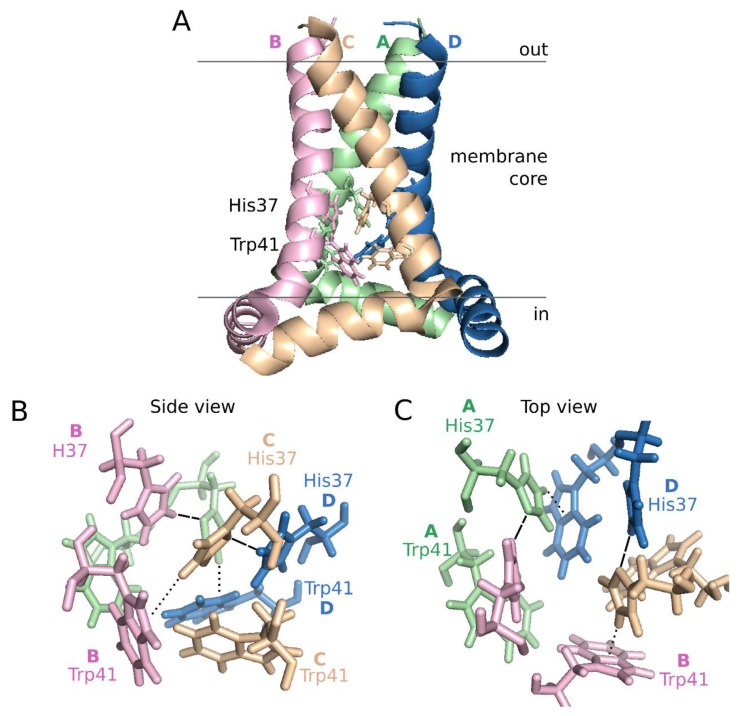
Influenza A M2 structure at pH 7.5. Schematic representation of the influenza A proton channel (Residues 22–62) based on the 2L0J structure [96]. A. Lateral view of the M2 tetramer. Monomers, from A to D are depicted in green, pink, pale orange and blue, respectively, using a cartoon representation. The side chains of His37 and Trp41 are highlighted in lines using the same color code. B and C. Side and top view representations of the HxxxW quartet, respectively. Dashed lines indicate hydrogen bonds, dotted lines cation-π interactions.

Structurally, both the full length M2 protein and its TM domain have been studied extensively using different techniques (i.e., SSNMR and X-ray crystallography) in various lipid mimetic enviroments [92,93,94,95,96]. Despite some differences, all the structures show a helical tetramer perpendicular to the membrane normal (Figure 5, panel A). The helix bundle leaves an aqueous pore lined by Val27, Ser31, Gly34, His37, Trp41, Asp44, and Arg45, which include all the hydrophilic residues present in the TM sequence. The motif responsible for proton conductance and tetramerization is the tetrameric His37-Trp41 cluster, referred as the HxxxW quartet (Figure 5). Interestingly, the hexamer formed by HCV p7 presents an internal pore which include, as in IAV M2, mostly hydrophilic residues (Ile6, Asn9, Ser12, Asn16, Trp21, Leu28, Arg35) [79].

At neutral pH, one proton is shared between the N ε2 of His37 from Chain/Helix A and the N δ1 of an adjacent His37 (helix B). Likewise, His37 from helix C forms a hydrogen bond with N δ1 of His37 on helix D forming a pair of dimers. At the same time, the His37 N ε2 of helix B interacts with the indole ring of the Trp41 residue in helix C and the His37 N ε2 of helix D with the Trp41 residue in helix A resulting in the dimer of dimers that constitute the close configuration of the channel (Figure 5, panels B and C). The position of His37 on helices A and C and subsequently the tetramer structure is further stabilized by a hydrogen bond between the protonated N δ1 of the His37 and its respective backbone carbonyl oxygen. Once in the low pH of the endosome, a hydronium ion attacks one of the His37-His37 dimers in the luminal side of the pore. At this point, the His37 becomes stabilized by a hydrogen bond with water at the newly exposed N δ1. Subsequently, the N ε2 proton is exposed to the aqueous environment on the C-terminal side, allowing its released to the viral interior. Once the proton is released, the closed state is restored. In each round, changes among the HxxxW quartet can be accomplished by small rotations of the His37 and Trp41 side chains. For a detailed explanation on proton conductance see [76,97].

Despite all the work with the M2 viroporin there are still controversies on its tetramerization process. In a recent work, Kawano *et al.* [98] propose that monomeric M2 protein first dimerize through interactions between the amphipatic helix of two monomers. No implication of the TM domain is required at this point. According to the authors, once formed, the dimer is stabilized by a disulfide bond between Cys17 and Cys19. The dimer is stable at neutral pH, however at acidic pH (eg. endosome) cation-π interactions between protonated His37 and Trp41 induce the formation of the tetramer.

In any case, the role of the M2 TM region in the tetramer formation is crucial. The final quaternary structure of M2 is not primarily driven by van der Waals forces (as expected for most TM proteins) but by inter-helical hydrogen bonds and cation-π interactions between residues facing the aqueous pore within (a type of interaction that might not be unique of M2). These residues are also necessary for the proton transfer activity of the protein, representing a beautiful example of functional linkage and highlighting the plasticity and multi-functionality of TM domains. 

## 6. Concluding Remarks

RNA virus viroporins constitute an interesting example of the two-stage membrane protein folding model. In a first step viroporins insert into the membrane individually, using a pathway determined by their specific sequence and structural characteristics. In a second step, viroporins homo-oligomerize forming higher order structures crucial for the porating activity. Viroporins have been previously classify into two classes according to the number of TM segments in the protein. However, this apparently simple classification contains highly interesting features [10]. A close analysis of their sequence characteristics reveals a different mechanism of membrane insertion (step I) and oligomerization (step II) for class I and II viroporins. These differences might arouse not solely form the type of pore formed but also from the distinct viral genome organizations. Since viroporins are emerging targets for drug design, to fully explore anti-viroporin compounds as potential therapeutical agents we must significantly increase our understanding of their mechanism of action, their route to the membrane and their oligomerization requirements.
